# Gesundheitsökonomische Bedeutung antimikrobieller Resistenzen

**DOI:** 10.1007/s00103-025-04061-1

**Published:** 2025-05-08

**Authors:** Christian Willy, Felix Bröcker

**Affiliations:** 1 Klinik für Unfallchirurgie und Orthopädie, Septisch-Rekonstruktive Chirurgie, Forschungs- und Behandlungszentrum Rekonstruktion von Defektwunden, Bundeswehrkrankenhaus Berlin, Scharnhorststraße 13, 10115 Berlin, Deutschland; 2 Idorsia (Berlin) Pharmaceuticals GmbH, Berlin, Deutschland

**Keywords:** Multiantibiotikaresistenz, MRE, Kosten, Todesfälle, Wirtschaft, MDR, AMR, Costs, Deaths, Economy

## Abstract

Die Verbreitung multiresistenter Erreger (MRE) stellt ein globales Problem dar, welches die öffentliche Gesundheit und die Wirtschaft vor signifikante Herausforderungen stellt. Prognostizierte Entwicklungen lassen den Schluss zu, dass die Zahl der MRE-assoziierten Todesfälle bis 2050 global signifikant ansteigen wird. In etwa 6 % der nosokomialen Infektionen in Deutschland sind MRE involviert, was einer Anzahl von 24.000 bis 36.000 MRE-Infektionen pro Jahr entspricht. Zusammen mit den ambulant erworbenen Infektionen durch MRE sind es jährlich etwa 54.500 Infektionen. In Deutschland wird die Zahl der unmittelbar MRE-bedingten Todesfälle auf 9700 pro Jahr geschätzt. Für die Zukunft ist in der stationären Versorgung vor allem mit einer signifikanten Zunahme der Carbapenem-resistenten Isolate von *Klebsiella pneumoniae* und *Acinetobacter baumannii* zu rechnen. Eine überschlagmäßige Abschätzung der gesamtwirtschaftlichen Mehrkosten einer MRE-Infektion bei stationärer Behandlung in Deutschland ergibt eine Höhe von ca. 27.000 €. Unter Berücksichtigung der wirtschaftlichen Konsequenzen entspricht dies auf Deutschland hochgerechnet einem jährlichen Betrag von ca. 4 Mrd. €. Die genannten Daten basieren in der Regel auf Schätzungen. Um auch Kostenbereiche wie Aufwendungen für die Rehabilitation sowie die Folgen des großflächigen Antibiotikaeinsatzes in der Tierhaltung adäquat berücksichtigen zu können, besteht erheblicher Forschungsbedarf. Um die Effektivität künftiger Maßnahmen zur Prävention und Eindämmung von Multiresistenzen adäquat evaluieren zu können, sind mehr valide Daten unerlässlich.

## Abstract (en)

The global dissemination of multi-resistant pathogens represents a significant challenge for public health and the economy, given the considerable difficulties it presents in terms of disease control and economic stability. It is projected that the number of deaths associated with multi-drug-resistant organisms (MDROs) could increase significantly worldwide by 2050. MDROs are responsible for approximately 6% of healthcare-associated infections, which equates to 24,000 to 36,000 MDRO infections per year in Germany. In total, this equates to approximately 54,500 infections per year, including those acquired by outpatients. In Germany, the number of deaths directly attributable to MDROs is estimated to be 9700 per year. It is anticipated that there will be a notable rise in the number of carbapenem-resistant isolates of *Klebsiella pneumoniae* and *Acinetobacter baumannii* in the future, particularly within the context of inpatient care. A rough estimate of the total additional economic costs of a MRE infection for inpatient treatment in Germany is approximately € 27,000 per case. When economic consequences are considered, the total cost to Germany is estimated to be approximately € 4 billion. It should be noted that the data presented in the studies are usually based on estimates. There is a considerable need for research in order to adequately account for cost areas such as expenses for rehabilitation and the consequences of the large-scale use of antibiotics in animal husbandry. However, more valid data are essential in order to adequately evaluate the effectiveness of future measures to prevent and contain multi-resistance.

„The health burden of infections due to bacteria with AMR in the EU/EEA population is comparable to that of influenza, tuberculosis and HIV/AIDS combined“ [1].

## Einleitung

Die in den vergangenen Jahren publizierten Surveillance-Berichte der Weltgesundheitsorganisation (WHO; [2]) und des Europäischen Zentrums für Krankheitsprävention und Kontrolle (ECDC; [3]) weisen auf eine enorme Dynamik hin, mit der sich die Empfindlichkeit klinisch relevanter, vor allem gramnegativer Bakterien gegenüber antimikrobiell wirksamen Substanzen verschlechtert. So melden in vielen WHO-Regionen nationale Surveillance-Systeme Resistenzraten von *K. pneumoniae* gegenüber Drittgenerations-Cephalosporinen von über 50 %. Auch wenn Deutschland hinsichtlich einer solchen Resistenzentwicklungsdynamik noch nicht zu den „Problemregionen“ zählt, zeigen die Daten des europäischen Resistenz-Surveillance-Systems EARS-Net und der Antibiotika-Resistenz-Surveillance (ARS) des Robert Koch-Instituts (RKI) auch in Deutschland einen stetigen Anstieg der Häufigkeit multiresistenter Erreger (MRE) über die vergangenen Jahre. Vor diesem Hintergrund sollte ein Überblick über die Auswirkungen von Infektionen mit MRE auf die Gesundheit der Bevölkerung und die mit diesen Infektionen verbundenen Gesundheitskosten bzw. Auswirkungen auf die gesamtwirtschaftliche Leistungsfähigkeit der Solidargemeinschaft erarbeitet werden.

## Globale Auswirkungen der MRE-Infektionen auf die Gesundheit

Einer aktuellen Schätzung zufolge wurden für das Jahr 2021 weltweit 4,71 Mio. Todesfälle (95 % Unsicherheitsintervall (UI) 4,00–5,55) mit bakterieller Multiantibiotikaresistenz in Verbindung gebracht, von denen 1,14 Mio. (95 % UI 1,00–1,28) direkt auf Infektionen mit MRE zurückzuführen seien [4]. Hierbei stützt sich die Studie auf eine Schätzung, die die durch bakterielle MRE verursachten Todesfälle und DALYs („disability-adjusted life years“) für 22 Pathogene, 84 Pathogen-Arzneimittel-Kombinationen und 11 Infektionssyndrome in 204 Ländern und Territorien von 1990 bis 2021 berücksichtigt und auf einer Vielzahl von Datenquellen, darunter Todesursachendaten, Krankenhausentlassungsdaten, Mikrobiologiedaten, Literaturstudien und Arzneimittelverkaufsdaten, basiert. Methicillin-resistenter *Staphylococcus aureus* (MRSA) und Carbapenem-resistente gramnegative Bakterien wie *K. pneumoniae* und *A. baumannii* zeigten hierbei die größte Zunahme bei Multiantibiotikaresistenz-bedingten Todesfällen. Die Sterblichkeit variierte stark je nach Region. In einigen Regionen, wie z. B. in West- und Zentraleuropa, gab es eine Abnahme zumindest für die unter 70-Jährigen, während in anderen Regionen, insbesondere in Südasien, mittlerer Osten, Nord- und Subsahara-Afrika, die MRE-bedingte Sterblichkeit für die Altersgruppen der über 49-Jährigen anstieg. Es wird geschätzt, dass im Jahr 2050 weltweit 1,91 Mio. Todesfälle direkt auf eine Multiantibiotikaresistenz zurückzuführen sein werden, mit insgesamt 8,22 Mio. Todesfällen, die mit ihr in Verbindung stehen. Hierbei werden die höchsten Multiantibiotikaresistenz-bedingten Mortalitätsraten für Südasien und Lateinamerika sowie die Karibik prognostiziert. Unter einem Szenario mit besserer Gesundheitsversorgung könnten bis 2050 weltweit 92 Mio. Todesfälle vermieden werden, während die Entwicklung neuer Medikamente gegen gramnegative Bakterien 11,1 Mio. AMR-Todesfälle verhindern könnte. Die Studie betont die Notwendigkeit von Infektionsprävention, Impfungen, der Minimierung eines unangemessenen Antibiotikaeinsatzes und der Forschung zu neuen Antibiotika [4].

Die genannten aktuelleren Ergebnisse unterscheiden sich somit von den im Jahr 2016 publizierten Zahlen des *Review on Antimicrobial Resistance*, das bis 2050 10 Mio. Todesfälle durch MRE prognostiziert hatte [5]. Die Verfasser hatten seinerzeit eingeräumt, dass die groben Schätzungen durch fundierte wissenschaftliche Arbeit untermauert werden sollten, dennoch trug die Publikation erheblich dazu bei, die Multiantibiotikaresistenz als eine der dringendsten Gesundheitsbedrohungen des 21. Jahrhunderts zu positionieren. Weitere Zahlen zur Schätzung der globalen Belastung durch MRE wurden dann 2022 (Datenbasis 2019) publiziert [6]. Diese Schätzung hatte ergeben, dass von den rund 8,9 Mio. Todesfällen durch bakterielle Infektionen im Jahr 2019 1,27 Mio. Todesfälle (95 % UI 0,911–1,71) auf Antibiotikaresistenzen zurückzuführen seien und 4,95 Mio. Todesfälle mit Antibiotikaresistenzen in Verbindung gebracht werden könnten. Auch wenn die älteren Daten dieser beiden Studien (2016, 2022) von den jüngsten Ergebnissen der Publikation aus dem Jahr 2024 abweichen, wird einheitlich unterstrichen, dass vor allem die Carbapenemresistenz bei gramnegativen Bakterien zu einer erheblichen Zunahme der Belastung geführt hat und die Multiantibiotikaresistenz eine zunehmende globale Gesundheitsherausforderung ist, die ein dringendes Eingreifen erfordert.

## Auswirkungen der MRE-Infektionen auf die Gesundheit in der Europäischen Union (EU) und im Europäischen Wirtschaftsraum (EWR)

MRE haben auch eine erhebliche negative Auswirkung auf die Gesundheit der EU-/EWR-Bürger und sind das führende Problem im Bereich der Infektionskrankheiten [7]. Zwischen 2005 und 2015 hat die Multiantibiotikaresistenz in der EU/im EWR zugenommen. Seinerzeit war dort fast jede fünfte Infektion auf antibiotikaresistente Bakterien zurückzuführen. In einigen Ländern, wie Rumänien und Griechenland, waren es sogar rund 40 % der Infektionen [1]. Tatsächlich variiert der Beitrag verschiedener MRE zur allgemeinen Gesundheitsbelastung stark zwischen den EU-/EWR-Ländern, was die Notwendigkeit von auf die Bedürfnisse der jeweiligen Länder zugeschnitten Präventions- und Kontrollstrategien unterstreicht. Die Hauptfaktoren für die länderspezifischen Unterschiede in der Belastung durch MRE sind die Art des Einsatzes von Antibiotika (Häufigkeit, Art, Dosis und Dauer, Implementierung eines *Antibiotic-Stewardship*-Konzeptes), die Verfügbarkeit der Antibiotika im klinischen Alltag, die Qualität der Krankenhausversorgung (Umsetzung der Hygieneregeln), einschließlich der Verfahren zur Infektionsprävention und -kontrolle, sowie die Art des Monitorings und der Dokumentation der MRE-Infektionen [1].

**Abb. 1:**
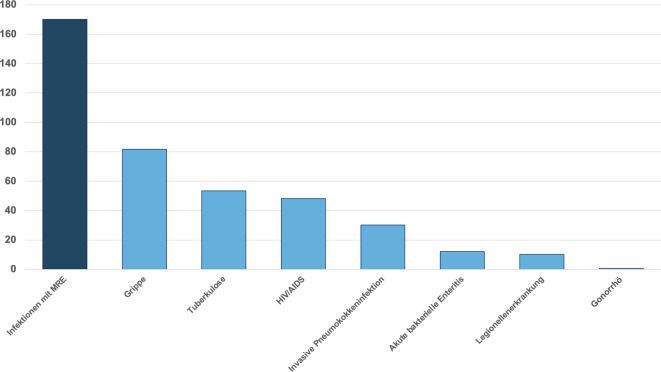
Gesundheitsbelastung durch multiresistente Erreger (*MRE*) und ausgewählte Infektionskrankheiten im Vergleich, in DALYs pro 100.000 Einwohner der Europäischen Union (*EU*) bzw. des Europäischen Wirtschaftsraumes (*EWR*). Daten für MRE-Infektionen: 2015, andere Infektionen: Durchschnitt 2009–2013 [9, 10]. (Abbildung in Anlehnung an [1]. *DALYs* Maß für die Quantifizierung der Krankheitsbelastung („disability-adjusted life years“), das nicht nur die Anzahl verlorener Jahre aufgrund vorzeitigen Todes, sondern auch die mit der Krankheit oder Behinderung gelebten Jahre bis zur Genesung oder zum Tod ausdrückt)

Die Organisation für wirtschaftliche Zusammenarbeit und Entwicklung (OECD) prognostiziert, dass die Multiantibiotikaresistenz in der EU/im EWR weiter zunehmen wird – von etwa 17 % der Infektionen mit AMR im Jahr 2015 auf 19 % im Jahr 2030. Ohne wirksame Gegenmaßnahmen wird die Antibiotikaresistenz gegen Zweit- und Drittlinienantibiotika in der EU/im EWR im Jahr 2030 um 72 % höher sein als noch 2005 [1, 8]. Schon die 2019 publizierte OECD-Kalkulation ergab für die EU und den EWR mehr als 670.000 Infektionen durch MRE als direkte Folge und 33.000 Todesfälle [1]. Herauszustellen ist, dass hierbei die Zahl der Todesfälle, die auf Infektionen mit *K. pneumoniae* zurückzuführen sind und bei denen das Bakterium gegen Carbapeneme – eine Gruppe von Reserveantibiotika – resistent war, um das 6‑Fache gestiegen ist; die Zahl der Todesfälle, die auf Infektionen mit *E. coli *beruhen, die gegen Cephalosporine der dritten Generation resistent sind, stieg um das 4‑Fache [1, 8]. Insgesamt ist die gesundheitliche Belastung durch Infektionen mit Bakterien, die gegen antimikrobielle Mittel resistent sind, in der EU-/EWR-Bevölkerung vergleichbar mit der von Influenza, Tuberkulose und HIV/Aids zusammen (Abb. 1; [1]). Bis 2050 werden AMR in den Ländern der EU und des EWR zu über 569 Mio. zusätzlichen Krankenhaustagen pro Jahr führen [1].

## Auswirkungen der MRE-Infektionen auf die Gesundheit in Deutschland

In Deutschland liegt der Anteil der Patienten, die sich während eines Krankenhausaufenthalts infizieren, bei etwa 3,6 % (mit oder ohne Antibiotikaresistenz), was schätzungsweise 400.000 bis 600.000 nosokomialen Infektionen pro Jahr entspricht [9–12].

Die häufigsten Arten nosokomialer Infektionen sind Harnwegsinfektionen, postoperative Wundinfektionen und Lungenentzündungen [13]. Basierend auf den Daten der Antibiotika-Resistenz-Surveillance (ARS) des RKI und der Prävalenzerhebung von 2011 wurden schätzungsweise ~6 % davon durch MRE verursacht, was 24.000 bis 36.000 Infektionen mit MRE pro Jahr in Deutschland entspricht [14]. Allein die 5 wichtigsten multiresistenten Erreger führen zu etwa 29.000 nosokomialen Infektionen (11.000 Infektionen durch MRSA, 4000 Infektionen durch Vancomycin-resistente Enterokokken (*Enterococcus faecalis* und *Enterococcus faecium*), 8000 Infektionen durch multiresistente *E. coli*, 2000 Infektionen durch multiresistente *K. pneumoniae* und etwa 4000 Infektionen durch multiresistente *P. aeruginosa*). Davon sind etwa 1500 Fälle, also 0,3 % aller nosokomialen Infektionen, auf MRE zurückzuführen, die gegen alle Antibiotikaklassen resistent sind („pandrug-resistant“; [9–12]). Prognosen zufolge erkranken in Deutschland jährlich infolge der auch ambulant erworbenen Infektionen etwa 54.500 Menschen an Infektionen durch MRE [15].

Im Rahmen einer 2019 veröffentlichten Studie des ECDC [3] zur Krankheitslast durch MRE (Schätzung für das Jahr 2015) wurde die Zahl der infektionsassoziierten Todesfälle für Deutschland auf ca. 2400 Menschen pro Jahr berechnet [16]. Anzumerken ist, dass die aufgeführten Zahlen nur Schätzungen sind. Hierdurch ist es erklärbar, dass 3 Jahre später ganz andere Zahlen genannt wurden. So formulierte der ehemalige Präsident des RKI, Lothar H. Wieler, im Oktober 2022 anlässlich der Einweihung des neuen WHO-Kooperationszentrums für Antibiotikaresistenz, -verbrauch und nosokomiale Infektionen am RKI, dass es in Deutschland „bis zu 9700 Todesfälle sind“, die direkt auf antimikrobielle Resistenz zurückzuführen seien [17], und 45.700 Todesfälle im Zusammenhang mit MRE [18]. Für die relevantesten Erreger beträgt die altersstandardisierte Sterblichkeitsrate im Zusammenhang mit der Multiantibiotikaresistenz pro 100.000: *E. coli:* 7,9; *S. aureus:* 4,0; *E. faecium:* 3,4; *K. pneumoniae:* 2,2 und *P. aeruginosa:* 1,6.

## Entwicklung der Resistenz in Deutschland

Im Hinblick auf die zukünftige Entwicklung von Antibiotikaresistenzen ist es notwendig, ein differenziertes Bild für die verschiedenen Erreger zu zeichnen. So zeigte sich basierend auf Daten der Deutschen Antibiotika-Resistenz-Surveillance (ARS) für *E. faecium* zwar zwischen 2014 und 2017 ein dramatischer Anstieg der Vancomycinresistenz von 11,2 % auf 26,1 % [19], diese Rate war aber in den letzten Jahren wieder leicht rückläufig und erreichte 2022 erstmals seit 2018 mit 18,1 % wieder einen Wert unter 20 % [20]. Für *S. aureus* wird ebenfalls eine sinkende Antibiotikaresistenz beobachtet. So sank der Anteil der MRSA-Isolate zwischen 2017 und 2022 von 12,5 % auf 7,1 % [21], was auf verbesserte Hygienemaßnahmen und gezielte Strategien zur Prävention zurückgeführt werden könnte. *K. pneumoniae* weist jedoch eine zunehmende Resistenzsituation auf. Während die Inzidenz der Carbapenemase-produzierenden *K.-pneumoniae*-Isolate in der COVID-19-Pandemie rückläufig war, zeigt sich seit März 2022 eine höhere Inzidenz als vor der Pandemie [22]. Auch bei *A. baumannii* ist ein Anstieg der Carbapenem-resistenten Isolate in der stationären Versorgung von 3 % im Jahr 2021 zu 5,9 % im Jahr 2022 zu verzeichnen, während das entsprechende Niveau für *P. aeruginosa* konstant bei 5,4 % lag [23]. Für *E. coli* wurde für den Zeitraum 2014–2020 ein leicht sinkender Anteil an Isolaten mit Resistenz gegen Cephalosporine der dritten Generation von 9,5 % auf 8,3 % beobachtet, während Fluorchinolonresistenzen bei etwa 20 % im gleichen Zeitraum nahezu konstant blieben [23]. Während also bei einigen der kritischen ESKAPE-Erreger (Akronym für ***E****. faecium*, ***S****. aureus*, ***K****. pneumoniae*, ***A****cinetobacter baumannii*, ***P****seudomonas aeruginosa* und ***E****nterobacterales*) Fortschritte erzielt wurden, nehmen die Resistenzen bei anderen deutlich zu.

Für die nahe und mittlere Zukunft ist in Deutschland und auch weltweit trotz dieser partiellen Erfolge mit einer starken Zunahme der Antibiotikaresistenzen zu rechnen. So wird geschätzt, dass der oben beschriebene Trend der annähernden Verdopplung der Todesfälle bis 2050 [4] auch für die Untergruppe der Länder mit hohem Einkommen, also auch für Deutschland, gilt. Der Anstieg ist vor allem auf die Verschiebung der Altersstruktur zurückzuführen. In der Altersgruppe der Über-70-Jährigen ist mit einer steigenden Anzahl von Infektionen mit MRE zu rechnen. Gleichzeitig wird deren Anteil an der Bevölkerung am stärksten steigen. Weitere Faktoren, die die Verbreitung von Antibiotikaresistenzen auch in Deutschland beschleunigen können, sind unter anderem der Klimawandel [24] und die mit der aktuellen Migration [25, 26], den Folgen der heutigen bewaffneten Konflikte [22, 27], aber auch die mit den zivilen Reiserückkehrern [28] verbundene geografische Verbreitung diverser multiresistenter Erreger. Zusätzlich wirkt sich der Antibiotikaeinsatz in der wachsenden industriellen Landwirtschaft ungünstig aus [29].

Bei all diesen Überlegungen darf nicht vergessen werden, dass die Multiantibiotikaresistenz nicht nur die Behandlung bestehender Infektionen erschwert, sondern auch die Therapie anderer Krankheiten beeinträchtigt [30]. So erfordert sie beispielsweise eine Modifikation der perioperativen Antibiotikaprophylaxe. Zudem erschwert sie die Durchführung von erfolgreichen Organtransplantationen, da sie die Patienten komplexeren Infektionen aussetzt und damit die Wahrscheinlichkeit eines Transplantatversagens und die postoperative Mortalität erhöht. Weiterhin wird die Therapie von Infekten vor einer Chemotherapie ohnehin immungeschwächter Patienten erschwert. Gleiches gilt für die im Rahmen einer onkologischen Chemotherapie auftretenden Infektkomplikationen [30].

## Globale Auswirkungen der MRE-Infektionen auf Gesundheitskosten und die Wirtschaft

Die mit Antibiotikaresistenzen verbundene Belastung wirkt sich nicht nur auf die Gesundheit des betroffenen Patienten aus (Mortalität oder Morbidität), sondern auch auf die Gesundheitsversorgung und Gesamtwirtschaft (Erwerbstätigkeit, Produktivität). Die Arbeitsgruppe „Review on Antimicrobial Resistance“ unter dem Vorsitz von Jim O’Neill hatte bereits 2016 erste Schätzungen der potenziellen zukünftigen MRE-Belastung veröffentlicht, die nur einen Teil der Auswirkungen von Antibiotikaresistenzen betrachteten. Die Arbeitsgruppe zeigte, dass eine anhaltende Zunahme der Resistenz bis 2050 zum Sinken des globalen Bruttoinlandsprodukts (BIP) um 2–3,5 % führen würde. Das bedeutet, dass die Welt bis 2050 mit einem Verlust an Wirtschaftsleistung im Wert von 60–100 Bill. US-Dollar rechnen muss, wenn die Resistenz gegen antimikrobielle Arzneimittel nicht erfolgreich bekämpft wird [5, 7, 31]. Andere Studien bezifferten die jährlichen Kosten bis 2050 weltweit auf zwischen 300 Mrd. und über 1 Bill. US-Dollar [30]. Für die Vereinigten Staaten wurde seitens der Centers for Disease Control and Prevention (CDC) geschätzt, dass die Kosten der Antibiotikaresistenz jedes Jahr 55 Mrd. US-Dollar betragen, davon 20 Mrd. US-Dollar für die Gesundheitsversorgung und etwa 35 Mrd. US-Dollar für Produktivitätsverluste [30].

Noch bedeutendere MRE-assoziierte Einbußen skizzieren Studien, die auch die globalen sozialen Auswirkungen von Produktivitätsverlusten versuchen zu beziffern. Ihnen zufolge hat die „Antibiotikakrise“ das Potenzial, die Kluft zwischen Entwicklungs- und Industrieländern zu vergrößern, was zu einer weiteren Zunahme der Ungleichheit führen würde. Die meisten Menschen, die durch Infektionen mit MRE in extreme Armut gedrängt werden könnten, stammen aus Ländern mit niedrigem Einkommen [32]. Die ohnehin schon benachteiligten Bevölkerungsteile der Welt würden am stärksten betroffen sein, da diese Länder stärker vom Arbeitseinkommen abhängig sind, das bei einer hohen Prävalenz von Infektionskrankheiten sinken würde. Jüngste Untersuchungen der Weltbank deuten darauf hin, dass die Multiantibiotikaresistenz die Armutsquote in Ländern mit niedrigem Einkommen im Vergleich zum Rest der Welt erhöhen wird [32]. Auch weitere Studien zeigen, dass das jährliche globale BIP um über 1 % sinken könnte, es aber in Entwicklungsländern bis 2050 zu einem Verlust von 5–7 % kommen würde [33]. Dieser Prozentsatz entspricht letztlich kumulierten 100–210 Bill. US-Dollar [7, 32]. Allein die multiresistente Tuberkulose (TBC) könnte für die Welt bis zum Jahr 2050 Kosten in Höhe von 16,7 Bill. US-Dollar verursachen [30].

## Europaweite Auswirkungen der MRE-Infektionen auf Gesundheitskosten und die Wirtschaft

Infektionen mit MRE haben in Europa ebenfalls eine negative Auswirkung auf die Budgets der Gesundheitssysteme sowie Auswirkungen auf die gesamtwirtschaftliche Leistung. So sind sie mit einem deutlichen Rückgang der Erwerbsbeteiligung und einer Verringerung der Produktivität verbunden, die z. B. bis 2050 in den 34 einbezogenen OECD- und EU/EWR-Ländern schätzungsweise mehr als 734.000 Vollzeitbeschäftigte pro Jahr betreffen wird. Diese Verluste sind in erster Linie auf den Rückgang der Beschäftigung sowie auf die Verringerung der Arbeitsproduktivität zurückzuführen, insbesondere aufgrund des erheblichen Anstiegs der Fehlzeiten am Arbeitsplatz [34]. Antimikrobielle Resistenzen verursachen in der EU und im EWR Jahr für Jahr geschätzte Kosten in Höhe von annähernd 11,7 Mrd. € (24 € pro Kopf). Davon entfallen 6,6 Mrd. € (etwa 56 %) auf Ausgaben für die Behandlung von Infektionen mit resistenten Erregern und ihren Folgen. Weitere 5,1 Mrd. € (etwa 44 %) entfallen auf wirtschaftliche Verluste infolge einer verringerten Erwerbstätigkeit, z. B. durch vorzeitige Todesfälle oder eine geringere Produktivität aufgrund langer krankheitsbedingter Fehlzeiten [3].

## Daten zu Kosten für MRE-Infektionen in Deutschland

Infektionen mit MRE verursachen auch in Deutschland erhebliche Kosten. In Infobox 1 werden Gründe für direkte und indirekte Kosten aufgelistet, wobei zu beachten ist, dass für die meisten indirekten Kosten die wissenschaftliche Evidenz derzeit noch fehlt.

Laut einer 9 Jahre alten Studie des Wissenschaftlichen Instituts der Techniker Krankenkasse (TK) betragen die Mehrkosten pro MRE-Infektion durchschnittlich 17.517,41 € (Auswertung zus. mit Universität Greifswald anonymisierter Daten von bundesweit 11.000 mit MRE infizierten TK-Versicherten; [35, 36]), wobei es nicht primäres Ziel dieser Analyse war, Aussagen über die MRE-bedingten volkswirtschaftlichen Gesamtkosten zu treffen. Die Kosten für die Krankenkassen setzten sich in dieser Studie wie folgt zusammen:

- Stationäre Behandlung: Durchschnittlich knapp über 20 zusätzliche Krankenhaustage (3-mal so lang wie die reguläre Verweildauer) führten zu Mehrkosten von etwa 16.230 € (92,6 % der Gesamtkosten).
- Ambulante Nachbehandlung: Nach dem Krankenhausaufenthalt entstehen Kosten von rund 100 €.
- Medikamentöse Therapie: Für die Bekämpfung der Infektion werden Medikamente im Wert von durchschnittlich zusätzlich 1187 € verordnet.

Die Autoren der genannten Studie stellten die Überlegung an, dass die realen Mehrkosten der Krankenversicherung, nicht über-, sondern eher unterschätzt werden, da viele bei dieser Zusammenstellung nicht betrachtete Kostenarten eine weitere Vergrößerung der Differenz zwischen MRE- und Kontrollgruppe bewirken. So könnte eine Verlängerung der Krankheitsdauer dazu führen, dass Kosten einer Haushaltshilfe, die laut § 38 SGB V unter bestimmten Voraussetzungen von der Krankenkasse zu bezahlen ist, höher ausfallen. Auch die Kosten für eventuell notwendige zusätzliche oder länger andauernde häusliche Krankenpflege wurden nicht einbezogen. Transferzahlungen, z. B. von Krankengeld, stellen zwar aus volkswirtschaftlicher Sicht keine Kosten dar, da ihnen kein Ressourcenverzehr gegenübersteht [19], sind aber aus Sicht der Krankenkassen Ausgaben. Die Kosten der Bereiche Rehabilitation, Heil- und Hilfsmittel wurden ebenfalls nicht in die Auswertung einbezogen [35]. Im Vergleich zu dieser 2015 publizierten Studie mit Einzelfallmehrkosten von 17.500 € waren in älteren Studien zuvor Kosten von 8887 € (Jahr 2003; [37]) und im Jahr 2005 Kosten von 12.895 € [38] genannt worden (fokussiert auf MRSA).

2018 zeigten Puchter et al., dass nosokomiale Infektionen durch Vancomycin-resistente Enterokokken (VRE) ebenfalls mit signifikant höheren Krankenhauskosten einhergehen [39].

Die medianen Gesamtkosten betrugen pro Fall 57.675 € für VRE-Infektionen im Vergleich zu 38.344 € für Vancomycin-sensitive Enterokokken-(VSE-)Infektionen (Differenz: 19.331 €). Die signifikantesten Kostenunterschiede (Hauptkostentreiber) zwischen VRE- und VSE-Patienten nach Infektionsbeginn bestanden bei Arzneimitteln (6030 € vs. 2801 €; = +215 %), Pflegepersonal (8956 € vs. 4621 €; = +194 %), medizinischen Produkten (3312 € vs. 1838 €; = +180 %) und medizinischen Assistenten (3766 € vs. 2474 €; = +152 %; [39]).

Nicht angesprochen ist bei all diesen Kostenzusammenstellungen (siehe auch Infobox 1), dass ein Teil der Mehrkosten der Krankenhäuser im aktuellen DRG-System (Diagnosis Related Groups) nicht vergütet werden und ebenso dass die Krankenhauskosten aus gesamtgesellschaftlicher Sicht aufgrund der dualen Finanzierung nicht dargestellt werden können (1. öffentliche Investitionskostenförderung nach § 2 Nr. 2 und 3 im Gesetz zur wirtschaftlichen Sicherung der Krankenhäuser und zur Regelung der Krankenhauspflegesätze – KHG, 2. Finanzierung der Betriebskosten nach § 2 Nr. 4, § 4 Nr. 2, §§ 16 ff KHG; [35]). Erwähnt werden muss, dass nosokomiale Infektionen mit MRE als Komplikation auch teilweise gegenfinanziert werden können. Obwohl Krankenhäuser im DRG-System für nosokomiale Infektionen durch höhere Schweregradstufen mehr Geld erhalten, kompensieren diese zusätzlichen Erlöse in der Regel nicht die gesamten durch die Infektionen verursachten Mehrkosten.

Ebenfalls nicht berücksichtigt sind in den oben genannten Berechnungen die finanziellen Mehrbelastungen in den Pflegeheimen. So zeigte die Arbeitsgruppe von Hübner et al. bereits 2016 in einer Analyse der Jahre 2011–2013, dass im Durchschnitt pro Pflegeheim 11,8 (SD ± 6,3) MRE-Fälle (Dauer pro Fall 163,3 ± 97,1 Tage) auftraten [40] und dies mit jährlichen MRE-bezogenen Mehrkosten zwischen 2449,72 € und 153.263,74 € bei einem Durchschnitt von 12.682,23 € pro Fall verbunden war. Die Hauptkostenfaktoren waren Personalkosten (43,95 € pro Tag und 7177,04 € pro Fall) und Kosten für Isolationsmaterial (24,70 € pro Tag und 4033,51 € pro Fall; [40]).

Eine grobe Schätzung der gesamtwirtschaftlichen Kosten von MRE-Infektionen in Deutschland basierend auf den in diesem Artikel enthaltenen Daten ist in Infobox 2 dargestellt. Insgesamt ergeben sich 4–4,5 Mrd. €, wobei nicht alle indirekten Kosten, wie z. B. jene im *One-Health*-Kontext, berücksichtigt werden konnten.

## Gesundheitsökonomische Aspekte unter Berücksichtigung des One-Health-Ansatzes

Obwohl konkrete Zahlen fehlen, ist davon auszugehen, dass MRE auch in der Veterinärmedizin zu signifikanten wirtschaftlichen Belastungen führen. So verursachen folgende Faktoren durch die Multiantibiotikaresistenz unter den Bedingungen eines One-Health-Ansatzes, der die Verbindung zwischen menschlicher Gesundheit, Tiergesundheit, Lebensmittelsicherheit und Umwelt berücksichtigt, erhebliche finanziellen Belastungen:

- Medikamentenkosten zur Behandlung,
- Tierarztkosten infolge der komplexeren Infektionen,
- Isolierungs- und Hygienemaßnahmen (Quarantäne oder Desinfektionsmaßnahmen),
- Produktionsverluste (weniger Milchproduktion, geringere Gewichtszunahme, Tierverluste),
- Präventionsmaßnahmen (Impfprogramme, Prophylaxemaßnahmen, verbesserte Hygiene- und Biosicherheitsmaßnahmen),
- MRE-bedingte Handelsrestriktionen bei Fleisch- und Tierprodukten,
- Imageverluste mit vermindertem Absatz,
- Überwachung (Monitoringprogramme),
- Forschungskosten (Entwicklung neuer Behandlungsansätze),
- Strategien zur Bekämpfung von multiresistenten Erregern,
- Gesundheitskosten im Humanbereich durch Zoonosen.

Eine sektorübergreifende Zusammenarbeit ist entscheidend, um die Ausbreitung von Antibiotikaresistenzen zu bekämpfen, und von großer Bedeutung, da der Großteil der Antibiotika in der Viehzucht eingesetzt wird – im Jahr 2010 waren es weltweit noch 63.200 t Antibiotika (erstes globales Mapping [41], 2020 einer Schätzung für 229 Länder zufolge bereits 99.500 t [42]).

Da wichtige Reserveantibiotika, die beim Menschen nur extrem zurückhaltend eingesetzt werden (wie das antimikrobielle Peptid Colistin), in der Tierhaltung – beispielsweise auf Geflügelhöfen [43] – als primäre und oft einzige Behandlungsmöglichkeit verwendet werden, bestehen erhebliche Risiken für die Übertragung antibiotikaresistenter Bakterien von Tieren auf Menschen durch Lebensmittel, direkten Kontakt oder gemeinsame Umweltquellen wie Abwasser [30]. Dies verdeutlicht die Komplexität der Multiantibiotikaresistenz in unserer modernen Welt und die Notwendigkeit eines umfassenden Ansatzes.

Einige allgemeine Schätzungen und Ansätze zur Einschätzung der hierdurch auftretenden ökonomischen Belastungen sind kaum verfügbar, sind jedoch ebenfalls in Milliardenhöhe zu veranschlagen. So zeigte eine Untersuchung der Europäischen Behörde für Lebensmittelsicherheit [44] und des Europäischen Zentrums für die Prävention und die Kontrolle von Krankheiten [3], dass antibiotikaresistente Bakterien jährlich Kosten in Milliardenhöhe verursachen, die nicht nur auf den Gesundheitssektor beschränkt sind, sondern auch indirekte Kosten für landwirtschaftliche Betriebe umfassen.

## Fazit

Die Multiantibiotikaresistenz verursacht jedes Jahr enorme finanzielle Schäden. Die Gesamtkosten im Zusammenhang mit Infektionen durch MRE in Deutschland sind jedoch nicht ausreichend genau zu beziffern. Die Analysen, auf denen die wenigen vorhandenen Zahlen basieren, beziehen eine Vielzahl von Aspekten, die sich finanziell nachteilig auswirken, nicht mit ein (siehe Infobox 1). Die bisherigen Bemühungen, diese Informationen bereitzustellen, resultierten in Schätzungen, die in vielen Fällen unzureichend und bisweilen widersprüchlich sind. Dieser Informationsmangel ist in erster Linie auf kleine Kliniksamples und Modellannahmen, Verzerrungen (Bias), eine suboptimale Studienqualität sowie die Verwendung inadäquater gesundheitsökonomischer Modellierungstechniken und unzweckmäßiger Methoden zur Ermittlung und Messung der Kosten zurückzuführen [45–50], wodurch keine zuverlässige Evidenz erarbeitet werden konnte [31]. Dennoch lassen bereits grobe Abschätzungen unter Berücksichtigung der hier dargelegten Zahlen den Schluss zu, dass die zusätzlichen Kosten auch in Deutschland erheblich sind (vgl. Infobox 2) und sich auf mehrere Milliarden Euro pro Jahr belaufen.

Vor dem Hintergrund der signifikanten volkswirtschaftlichen Belastung und der Tatsache, dass Antibiotikaresistenzen weltweit eine der häufigsten Todesursachen sind (häufiger als HIV/Aids oder Malaria), erscheint es unverständlich, dass die Gesundheitssysteme und Kliniken auch nach nahezu 2 Jahrzehnten wissenschaftlicher Problemanalyse keine ausreichend verfeinerten Berechnungen der zurechenbaren Kosten und gesundheitlichen Auswirkungen von arzneimittelresistenten Mikroorganismen besitzen. Valide Daten sind jedoch dringend erforderlich, um die Effizienz aktueller Maßnahmen zur Prävention und Eindämmung von Multiantibiotikaresistenzen zu bewerten und fundierte Entscheidungen für zukünftige Initiativen zur Bekämpfung der globalen Bedrohung durch MRE zu treffen.

### Infobox 1 Gründe für direkte und indirekte Kosten durch Infektionen mit multiresistenten Erregern (MRE)

Die Gesundheitsbudgets in den OECD- und EU/EWR-Ländern werden durch Infektionen mit MRE erheblich belastet. Die Behandlung von Infektionen, die durch antimikrobiell resistente Erreger verursacht werden, ist im Vergleich zu Infektionen, die durch empfindliche Erreger verursacht werden, wesentlich kostspieliger [51, 52]. Gründe* sind:

- intensivere medizinische Verfahren und teurere MRE-spezifische Therapiekosten (Einsatz von Zweitlinienbehandlungen oder aufwendigere Kombinationen von antimikrobiellen Mitteln),
- zusätzliche und umfassendere Untersuchungen (z. B. Labortests, mikrobiologische Untersuchungen, zudem mikrobiologisches Screening von Patienten, Kosten für die Überwachung des Antibiotikaspiegels),
- längere Krankenhausaufenthalte,
- Opportunitätskosten (z. B. Bettensperrung, die bei mancher Berechnung der zusätzlichen Kosten mit 77,45 % den weitaus größten Anteil ausmacht; [53]),
- Kosten des Hygienemanagements (z. B. Isolierungsmaßnahmen auch bei nur kontaminierten Patienten, Vernichtung/Aufbereitung von Materialien/Wäsche, abschließende Raum- und Instrumentenreinigung, Patiententransport, Ein- und Ausschleusmaßnahmen während stationären Aufenthaltes, Eradikationsmaßnahmen),
- erhöhte Materialkosten,
- erhöhter Zeitaufwand des Personals (inkl. der Zeit zur Personalschulung),
- Auswirkungen auf die Motivationslage der Pflegemitarbeiter infolge erhöhter Belastung; der zusätzliche Arbeitsaufwand (Isolationsmaßnahmen; Tragen von Schutzkleidung und das Einhalten strenger Hygienerichtlinien; erhöhter Zeitaufwand) und erhöhte emotionale Belastung können Erschöpfung und Frustration bei Pflegekräften hervorrufen (v. a. wenn Ressourcen knapp sind; Sorge um Eigen- und Patientenschutz; erhöhte Verantwortung),**
- ambulante Nachbetreuung,
- Kosten einer Haushaltshilfe (ggf.),
- zusätzliche oder länger andauernde häusliche Krankenpflege,
- Transferzahlungen, z. B. Krankengeld,
- Kosten der Bereiche Rehabilitation, Heil- und Hilfsmittel,
- zusätzliche Kosten für die Angehörigen (ggf.),
- langfristige gesundheitliche Folgen der Patienten (chronische Erkrankungen, wiederkehrende Infektionen, Notwendigkeit weiterer medizinischer Behandlungen), die Lebensqualität und Arbeitsfähigkeit mindern,
- Kosten durch Produktivitätsverluste und durch Arbeitsausfälle (verminderte Erwerbsfähigkeit),
- Kosten der Multiantibiotikaresistenz unter den Bedingungen eines *One-Health*-Ansatzes (Berücksichtigung der Auswirkungen auf die Tiergesundheit, Lebensmittelsicherheit und die Umwelt).

* *Aufschlüsselung der indirekten Kosten*: Aufgrund fehlender Studien besteht für die genannten potenziellen indirekten Gründe erhöhter Kosten meist keine wissenschaftliche Evidenz. Sie sollen zum besseren Verständnis der Komplexität dieser Problematik dennoch aufgeführt sein.

** *Auch positive Auswirkungen sind denkbar*: Pflegekräfte, die sehen, dass ihre Arbeit dazu beiträgt, Infektionen zu verhindern und Patienten zu helfen, können darin eine Quelle von Erfüllung und Sinnhaftigkeit finden. Dies kann die Motivation steigern, trotz der zusätzlichen Herausforderungen. Somit hat die Behandlung von MRE-Patienten sowohl belastende als auch potenziell motivierende Aspekte. Die Auswirkungen auf die Motivationslage der Pflegemitarbeiter hängen stark davon ab, wie gut sie unterstützt werden, welche Ressourcen ihnen zur Verfügung stehen und wie sie ihre eigene Rolle und den Umgang mit diesen Herausforderungen wahrnehmen.

### Infobox 2 Grobe Abschätzung der gesamtwirtschaftlichen Kosten der Multiantibiotikaresistenz (MRE) am speziellen Beispiel Deutschland

Die überschlagmäßige Abschätzung basiert auf den im vorliegenden Artikel genannten Zahlen. Unter Berücksichtigung dieser Daten wird konservativ von 40.000 (im Vgl. zu bis zu 36.000 in den Jahren 2015–2017 [9]) stationär zu behandelnden klinisch relevanten nosokomialen Infektionen mit MRE im Jahr 2025 ausgegangen. Zudem ist zwischen 2018 und 2025 mit einer signifikanten Steigerung der Gesundheitskosten in Deutschland von 391 Mrd. € auf geschätzt 550 Mrd. € (+ ca. 41 %) zu rechnen. Die Mehrkosten für eine MRE-Infektion bei stationärer Behandlung betragen somit 27.218 € (+ 41 % gegenüber 19.331 € [39] im Jahr 2018). Allein dieser Kostenpunkt stellt eine Belastung von ca. 1,1 Mrd. € (40.000 × 27.218 €) dar.

Unter Berücksichtigung der Tatsache, dass die vorliegende Abschätzung nur die zusätzlichen Ausgaben für die Behandlung von Infektionen mit resistenten Erregern umfasst (somit etwa 56 % aller Kosten [3]) und daher 44 % für die wirtschaftlichen Verluste infolge der verringerten Erwerbstätigkeit hinzugerechnet werden müssen (ECDC, 2023, S. 161), lässt sich eine Gesamtsumme von 1,94 Mrd. € ableiten.

In derselben Quelle [3] wurde eine Schätzung der jährlichen Kosten infolge antimikrobieller Resistenzen pro Kopf in Höhe von 24 € (2023) veröffentlicht. Bei einer Veranschlagung von 25 € für jeden Einwohner Deutschlands im Jahr 2025 entspricht dies einer jährlichen Summe von 2,1 Mrd. €.

In diesem Kontext ist zudem der Betrag zu berücksichtigen, der außerhalb von Krankenhäusern in Pflegeheimen bei der nicht mehr erwerbsfähigen Bevölkerung entsteht. Im Rahmen einer Erhebung, die in den Jahren 2011 bis 2013 in verschiedenen Pflegeheimen durchgeführt wurde, konnte eine durchschnittliche Anzahl von Fällen einer MRE-Infektion von 11,8 pro Pflegeheim ermittelt werden. Die durchschnittlichen MRE-assoziierten Kosten pro Fall betrugen 12.682,23 € [40]. Unter Berücksichtigung einer seit 2013 bestehenden Kostensteigerung im Pflegesektor von geschätzt nur 30 % belaufen sich die Kosten im Jahr 2025 auf 14.331 €. Im Jahr 2021 gab es in Deutschland insgesamt 16.115 Pflegeheime [40]. Bei einer angenommenen Anzahl von 10 Patienten mit einer MRE-Infektion pro Pflegeheim und Jahr ergibt sich daraus eine zusätzliche Belastung in einer Größenordnung von ca. 2,3 Mrd. € (16.115 × 10 × 14.331 €).

In der Summe von somit ca. 4–4,5 Mrd. € sind jedoch beispielhaft nicht berücksichtigt:

- Kosten der Bereiche Rehabilitation, Heil- und Hilfsmittel,
- langfristige gesundheitliche Folgen der Patienten, die die Lebensqualität und Arbeitsfähigkeit durch Beeinträchtigungen mindern (chronische Erkrankungen, wiederkehrende Infektionen, Notwendigkeit weiterer medizinischer Behandlungen),
- Auswirkungen auf die Motivationslage der Pflegemitarbeiter und deren Effekte,
- Kosten der Multiantibiotikaresistenz unter den Bedingungen eines *One-Health*-Ansatzes.
